# Quantification of human bocavirus in lower respiratory tract infections in China

**DOI:** 10.1186/1750-9378-2-3

**Published:** 2007-01-31

**Authors:** Feng Lin, Aiping Zeng, Ningmin Yang, Haiyan Lin, En Yang, Shengqi Wang, David Pintel, Jianming Qiu

**Affiliations:** 1 Wenling First Hospital, Wenling, Zhejiang Province, China; 2 Hangzhou Zhiyuan Institute of Medical Diagnostics, Hangzhou, China; 3 Beijing Institute of Radiation Medicine, Beijing, China; 4 Department of Molecular Microbiology and Immunology, University of Missouri-Columbia, Columbia, MO, USA; 5 Department of Microbiology, Molecular Genetics and Immunology, University of Kansas Medical Center, Kansas City, KS, USA

## Abstract

A quantitative PCR method was established to quantify human bocavirus (HBoV) genomic copies in clinical specimens from children with lower respiratory tract infections (LRTI) in China. A total of 257 respiratory tract specimens were tested, and 7 (2.7%) of these (all sputum samples) were positive, with genomic copies that ranged from 8.0 × 10^3 ^to 8.0 × 10^9 ^in the samples. The main clinical symptom of patients who were positive for HBoV DNA was a pneumonia-like syndrome represented by high fever and cough. Our results suggest that HBoV may be an important etiological agent of LRTI in children in China.

## Finding

Virus infection is the major cause of lower respiratory tract infections (LRTI) in children worldwide, and the most important viral agent is arguably respiratory syncytial virus (RSV) [1]. Others viruses such as influenza viruses, parainfluenza viruses, adenoviruses, rhinoviruses, coronaviruses, and human metapneumovirus are also frequently reported to cause LRTI [2-5]. Human bocavirus (HBoV) was first cloned from pooled human respiratory tract samples collected in Sweden, and was provisionally classified into the genus *Bocavirus *based on sequence comparisons [6]. HBoV has been reported worldwide to be present in between 1.5% to 11.3% of respiratory samples tested from individuals with acute respiratory illness [7-10], and it appears to be associated with LTRI [11-13]. To date, there have been no studies reporting the detection of HBoV DNA in children with LRTI from China. Currently, detection of HBoV in children with LRTI mainly relies on DNA amplification by regular PCR. Because these assays are not quantitative, positive results are hard to interpret. Recently, a reliable quantitative PCR (Q-PCR) method has been developed to detect HBoV genomic copies in clinical samples, and this has demonstrated a presence of HBoV DNA in children with pneumonia-like symptoms in Thailand [14]. In this study, we used a Q-PCR with the amplicon targeted to the NS coding region of HBoV to detect the presence of HBoV DNA in children with respiratory tract infection in China. Our results suggest that HBoV may be an important etiological agent of LRTI in children in China.

A total of 257 specimens were collected from December, 2005 to February, 2006 from infants or children with LRTI hospitalized in Wenling First Hospital, Zhejiang Province, China. The specimens included throat swab, nasopharyngeal aspirate, sputum and aspirated sputum, together with blood samples, on the day of hospitalization. All the blood samples tested negative for antibodies directed against influenza virus A and B, respiratory syncytial virus (RSV), parainfluenza virus and adenovirus by commercially available ELISA kits. All these clinical samples were taken after informed consent was obtained from parents or other legal guardians,

DNA extraction from clinical specimens was performed as follows. Throat swab and nasopharyngeal aspirates were diluted in 2 ml of PBS, and were centrifuged at 12,000 rpm at 4°C for 10 min. The pellets were resuspended in 200 μl PBS. The sputum and aspirated sputum specimens were digested with 3 volumes of 4% NaOH, and were centrifuged at 8,000 rpm at 4°C for 5 min. Pellets were further washed with PBS and resuspended in a final volume of 200 μl PBS. All these resuspended pellets were extracted DNA using QIAamp blood mini kit (QIAGEN). A plasmid (pskHBoV) containing the HBoV sequence (nts 1-5299) was synthesized by extension of PCR fragments with primers designed based on the ST2 sequence of HBoV [Genbank: DQ000496], and this was subsequently inserted into pBluescript vector (Stratagene). This plasmid was used as a control (1 genomic copy = 5 × 10^-12 ^μg) to establish the standard curve for absolute quantification using TaqMan technology with an Applied Biosystems 7500 system (Foster City, Calif.) as a quantitative PCR method [15,16]. The amplicon and the TaqMan probe for this HBoV specific quantitative PCR were designed by Primer Express 2.0.0 software(Applied Biosystems) in the conserved regions of the NS coding region among HBoV genome sequences deposited in GenBank. Their sequences are as follows: forward primer, 5' AGC TTT TGT TGA TTC AAG GCT ATA ATC (HBoV nts 1417 to 1444); reverse primer, 5' TGT TTC CCG AAT TGT TTG TTC A'3 (HBoV nts 1500 to 1480); and the probe, 5'FAM-TCT AGC CGT TGG TCA CGC CCT GTG-TAMRA3' (HBoV nts 1446 to 1469). TaqMan universal PCR master mix (Applied Biosystems) was used for amplification with the standard protocol. 5 μl of extracted DNA was used in a reaction volume of 25 μl.

HBoV DNA was detected in sputum and aspirated sputum. A total of 7 (2.7%) of 257 specimens tested were positive for HBoV by Q-PCR (Table 1). All 7 positive samples were either sputum or aspirated sputum, indicating a significant presence of HBoV DNA in lower respiratory tract. There was no significant age difference in detection of HBoV genome in specimens. These positive samples contained a substantial titer of virus with genomic copies ranged from 4.0 × 10^3 ^to 4.0 × 10^9^/ml in the original collected sample (2 ml), suggesting active replication of virus in the lower respiratory tract. The main clinical symptom of patients who were positive for HBoV DNA was a pneumonia-like syndrome represented by high fever (>39°C) and cough.

**Table 1:** Clinical characteristics of 7 patients positive for HBoV DNA by Q-PCR

No. specimens	Type of samples	Sex	Age	Symptom	Genomic copies (gc/ml)*	Clinical Diagnosis
WL102	Aspirated sputum	F	13 mo	Fever with cough for 10 day	1.46 × 10^7^	Pneumonia
WL108	Aspirated sputum	F	1 yr	Fever with cough for 5 day, seizure once	3.95 × 10^9^	Pneumonia
WL109	Sputum	F	7 yr	Fever for 2 days,	4.01 × 10^3^	Bronchiolitis
WL160	Aspirated sputum	M	7 mo	Fever for 5 days and cough for 3 days	4.51 × 10^3^	Bronchitis
WL221	Aspirated sputum	M	2 yr	Fever and cough for 2 days	6.95 × 10^5^	Bronchiolitis
WL223	Sputum	F	3 yr	Fever for 3 days	6.94 × 10^5^	Bronchiolitis
WL226	Sputum	M	3 yr	Fever and cough for 5 days	5.45 × 10^4^	Bronchitis

*The number of genomic copies was converted to per ml in the original collected sample (2 ml).

The rate of detection that we observed in this population was lower than the average of these previously reported [7-10]. This might reflect either a truly lower frequency of HBoV infection the Zhejiang Province of China, or, alternatively, variations in the amplicon within these Chinese HBoV isolates. In addition, lower copy numbers of viral genomes were not detected in our assay, perhaps because of limitations in the extraction of DNA from specimens or inhibitors of the Q-PCR in the clinical specimens. Further investigation with different paired primers and probes to quantify the HBoV genome in clinical samples are under way for probing the etiology of HBoV in LTRI in children, using, in addition, normal controls. Nevertheless, this quantitative PCR method provides a reliable means to screen samples with high titers of genomic copies for virus isolation and to begin to address the relationship between HBoV and LRTI in children in China.

## Abbreviations

HBoV (Human bocavirus); LTRI (Lower respiratory tract infection); Q-PCR (Quantitative polymerase chain reaction).

## Competing interests

The author(s) declare that they have no competing interests.

## Authors' contributions

FL planned the study, collected clinical samples in conformation with all the human subject consents and participated in work of ELISA, DNA extraction and Q-PCR performance. AZ and HL performed ELISA, extracted DNA and Q-PCR. NY, EY and SW developed and standardized the Q-PCR method. DP and JQ coordinated the study, constructed plasmid, designed primers and probe, initiated Q-PCR and wrote the manuscript.

## References

[B1] Iwane MK, Edwards KM, Szilagyi PG, Walker FJ, Griffin MR, Weinberg GA, Coulen C, Poehling KA, Shone LP, Balter S, Hall CB, Erdman DD, Wooten K, Schwartz B (2004). Population-based surveillance for hospitalizations associated with respiratory syncytial virus, influenza virus, and parainfluenza viruses among young children. Pediatrics.

[B2] Choi EH, Lee HJ, Kim SJ, Eun BW, Kim NH, Lee JA, Lee JH, Song EK, Kim SH, Park JY, Sung JY (2006). The association of newly identified respiratory viruses with lower respiratory tract infections in Korean children, 2000-2005. Clin Infect Dis.

[B3] Madhi SA, Kuwanda L, Cutland C, Klugman KP (2006). Five-year cohort study of hospitalization for respiratory syncytial virus associated lower respiratory tract infection in African children. J Clin Virol.

[B4] van den Hoogen BG, de Jong JC, Groen J, Kuiken T, de GR, Fouchier RA, Osterhaus AD (2001). A newly discovered human pneumovirus isolated from young children with respiratory tract disease. Nat Med.

[B5] van der HL, Pyrc K, Jebbink MF, Vermeulen-Oost W, Berkhout RJ, Wolthers KC, Wertheim-van Dillen PM, Kaandorp J, Spaargaren J, Berkhout B (2004). Identification of a new human coronavirus. Nat Med.

[B6] Allander T, Tammi MT, Eriksson M, Bjerkner A, Tiveljung-Lindell A, Andersson B (2005). Cloning of a human parvovirus by molecular screening of respiratory tract samples. Proc Natl Acad Sci U S A.

[B7] Foulongne V, Olejnik Y, Perez V, Elaerts S, Rodiere M, Segondy M (2006). Human bocavirus in French children. Emerg Infect Dis.

[B8] Bastien N, Brandt K, Dust K, Ward D, Li Y (2006). Human Bocavirus infection, Canada. Emerg Infect Dis.

[B9] Sloots TP, McErlean P, Speicher DJ, Arden KE, Nissen MD, Mackay IM (2006). Evidence of human coronavirus HKU1 and human bocavirus in Australian children. J Clin Virol.

[B10] Ma X, Endo R, Ishiguro N, Ebihara T, Ishiko H, Ariga T, Kikuta H (2006). Detection of human bocavirus in Japanese children with lower respiratory tract infections. J Clin Microbiol.

[B11] Simon A, Groneck P, Kupfer B, Kaiser R, Plum G, Tillmann RL, Muller A, Schildgen O (2006). Detection of bocavirus DNA in nasopharyngeal aspirates of a child with bronchiolitis. J Infect.

[B12] Manning A, Russell V, Eastick K, Leadbetter GH, Hallam N, Templeton K, Simmonds P (2006). Epidemiological profile and clinical associations of human bocavirus and other human parvoviruses. J Infect Dis.

[B13] Kesebir D, Vazquez M, Weibel C, Shapiro ED, Ferguson D, Landry ML, Kahn JS (2006). Human bocavirus infection in young children in the United States: molecular epidemiological profile and clinical characteristics of a newly emerging respiratory virus. J Infect Dis.

[B14] McIntosh K (2006). Human bocavirus: developing evidence for pathogenicity. J Infect Dis.

[B15] Lu X, Chittaganpitch M, Olsen SJ, Mackay IM, Sloots TP, Fry AM, Erdman DD (2006). Real-time PCR assays for detection of bocavirus in human specimens. J Clin Microbiol.

[B16] Qiu J, Cheng F, Burger LR, Pintel D (2006). The transcription profile of Aleutian mink disease virus in CRFK cells is generated by alternative processing of pre-mRNAs produced from a single promoter. J Virol.

[B17] Qiu J, Cheng F, Pintel DJ (2006). Expression profiles of bovine adeno-associated virus and avian adeno-associated virus display significant similarity to that of adeno-associated virus type 5. J Virol.

